# Much ado about something: a response to “COVID-19: underpowered randomised trials, or no randomised trials?”

**DOI:** 10.1186/s13063-021-05755-y

**Published:** 2021-11-07

**Authors:** Noah A. Haber, Sarah E. Wieten, Emily R. Smith, David Nunan

**Affiliations:** 1 Meta-Research Innovation Center at Stanford (METRICS), Stanford University, 1265 Welch Rd Palo Alto, Stanford, CA 94305 USA; 2 Department of Global Health, Milken Institute School of Public Health, George Washington University, 950 New Hampshire Avenue, Washington, DC 20052 USA; 3 Centre for Evidence Based Medicine, University of Oxford, Radcliffe Primary Care Building, Radcliffe Observatory Quarter, Woodstock Road, Oxford, OX2 6GG UK

**Keywords:** Non-pharmaceutical interventions, Masks, Ethics, DANMASK-19, Statistical power

## Abstract

Non-pharmaceutical interventions (NPI) for infectious diseases such as COVID-19 are particularly challenging given the complexities of what is both practical and ethical to randomize. We are often faced with the difficult decision between having weak trials or not having a trial at all. In a recent article, Dr. Atle Fretheim argues that statistically underpowered studies are still valuable, particularly in conjunction with other similar studies in meta-analysis in the context of the DANMASK-19 trial, asking “Surely, some trial evidence must be better than no trial evidence?” However, informative trials are not always feasible, and feasible trials are not always informative. In some cases, even a well-conducted but weakly designed and/or underpowered trial such as DANMASK-19 may be uninformative or worse, both individually and in a body of literature. Meta-analysis, for example, can only resolve issues of statistical power if there is a reasonable expectation of compatible well-designed trials. Uninformative designs may also invite misinformation. Here, we make the case that—when considering informativeness, ethics, and opportunity costs in addition to statistical power—“nothing” is often the better choice.

## Background

In a recent commentary “COVID-19: underpowered randomised trials, or no randomised trials,” Dr. Fretheim asks that “Surely, some trial evidence must be better than no trial evidence?” [1] when faced with the common decision of whether to conduct an underpowered trial or no trial at all. The commentary uses the example of DANMASK-19 [2, 3] trial to suggest that for public health and non-pharmaceutical interventions (NPIs)—where we rarely have large, well-powered trials upon which to base decisions—well-conducted but underpowered trials can both demonstrate the feasibility of larger trials and contribute toward a body of literature that can help reduce uncertainty. We agree that we must often face difficult choices when well-powered, well-designed, and well-conducted trials are not available. However, it is not a given that all trials contribute meaningfully toward reducing uncertainty or informing some hypothetical decision(s), either individually or collectively as part of the body of evidence. Uninformative trials—such as we argue is the case for DANMASK-19—can have harms [4]. Here, we make the case that when considering informativeness, ethics, and opportunity costs in addition to statistical power, “nothing” is often the better choice.

## Main text

Implementing a well-designed trial for NPIs requires facing a different set of challenges compared to pharmaceutical interventions. For example, policy interventions require that the unit of randomization be government units and institutions rather than individuals. To do such a trial requires large numbers of observable units to comply with the policy orders of an external research organization and likely requires vast social, political, and logistical coordination. While the recently released early results of a 300,000 person, 600 village cluster RCT on the impact mask-related interventions for COVID-19 in Bangladesh [5, 6] demonstrates that such trials may be feasible, it also demonstrates the complex combination of strong design, social buy-in, resources, infrastructure, and circumstances required to achieve it. Compliance—both at the intervention and/or individual level—is a key component for NPIs and often requires behavioral elements, like distancing and mask usage, but is notoriously difficult to ensure or measure. NPIs are also extremely heterogeneous and conditional on the specific settings in which they take place, posing difficulties for generalizability. Even stepped-wedge designs, often used for intervention roll-outs over time, are challenging when the outcome is an infectious disease [7] such as COVID-19; infectious disease takes time to spread through a population, outcomes may not manifest for weeks or months, and outcomes are highly subject to spillovers, population conditions, and complex disease dynamics over time. These issues and others mean that NPIs are particularly challenging to study with randomization or any other impact evaluation design.

Beyond statistical power, what is feasible to randomize and measure is often not informative for what we want to know. The DANMASK-19 authors defend their study as being a pragmatic trial [8], but having pragmatic limitations is not the same as designing a pragmatic trial [9]. It was not feasible to randomize mask *wearing* in the DANMASK-19 trial, nor was it feasible to measure community spread. In the DANMASK-19 trial, the main intervention was *messages* about mask wearing; it only measured infections for the employees enrolled in the trial rather than transmission to others (source control—arguably a more relevant concern for policy recommendations) and did not meaningfully measure the impact of messages on mask-wearing behavior.^1^ As a trial about the impact of messages, this trial was underpowered and poorly measured at the outset of its design. As a trial about the impact of mask *wearing*, it risks being severely biased toward the null due to compliance and testing [10]. Neither of the above interpretations meaningfully inform any decision.

While meta-analysis can often resolve issues of power as Dr. Fretheim notes, that is only true when there is a reasonable expectation that enough sufficiently compatible, well-designed, and well-executed trials will come into existence to collectively power one or more meta-analyses. While we do not need to be held back by arbitrary power thresholds [11], we must consider how much information our trials actually provide and at what expense [12]. That is a difficult value proposition for NPI trials, which often must deal with high degree of heterogeneity in the population, intervention, comparison group, and outcomes. No two “stay-at-home” mandates were the same, nor would we expect trials of them to be. At the very least, this heterogeneity increases meta-analysis sample sizes. At worst, it can render studies incompatible for comparison. In the case of DANMASK-19, it is difficult to imagine circumstances aligning to allow for many trials of comparable design for meta-analysis be feasible. Uncoordinated and underpowered trials lead to research waste [13]. Meta-analysis relies critically on the design strengths and weaknesses of its constituent parts; a meta-analysis of poorly designed studies is a poorly designed meta-analysis. 

Note that our argument does not rely on the use of null-hypothesis significance testing, whether through p-values or other metrics, nor do we consider it the most relevant metric. Statistical power is important under any decision-making paradigm, whether value for information-based frameworks, confidence interval decision thresholds, or others. The most important component of the evidentiary strength of a given study is in its design, rather than post-hoc measurements of uncertainty that result from its execution. Some of us expressed our concerns about DANMASK-19’s design issues before any results were released or available, but unfortunately after the trial had already been completed. Notably, our concern applies to the decision for whether to carry out a study a priori at the planning stages, not for publication considerations. Once a trial is conducted, in general it should be published in such a way to make its limitations well understood, regardless of the results.

If it is questionable that either individually or collectively, a hypothetical NPI trial would be informative, then we also must consider the ethical considerations for the trial participants. Why enroll participants, risk personal data, etc. for a trial that has little hope of being usefully informative and, therefore, little hope of providing social value? [14]. For NPIs, that is further complicated by the fact that individuals often cannot practically consent for group-level interventions and individual consent requirements can be waived only in some contexts [15]. Sorting out which consent requirements apply for a particular NPI study, even when well designed and adequately powered, can be complex. Beyond the participants, we must also consider the ethical ramifications to researchers, policy-makers, consumers, and those who might be impacted by decisions made due to poorly designed or underpowered research.

Randomized trials typically require large investments in coordination, funding, time, and other resources. Underpowered and poorly designed trials come without much hope of useful information in exchange for the expense. These are opportunity costs; we must also consider the value that could be achieved investing those resources elsewhere, including in different areas of research or in non-research investments. Resources for research are often not zero-sum, but they are always limited.

Underpowered and poorly designed trials are often worse than uninformative; they can be actively *mis*informative. Understanding why the DANMASK-19 trial was not suitably designed or powered to be informative for any actionable question requires a deep understanding of issues around statistical power, null hypothesis significance testing, and study design, as well as the time to dive into it. Without that, the DANMASK-19 trial invites misinterpretation, particularly with regard to fallacious interpretation of statistical insignificance. An uninformative trial that also invites misinterpretation can only have negative information gain, leading to misinformation for policy makers, researchers, journalists, and research consumers. Dr. Fretheim provides an example with misinformative headlines from the *Daily Mail*, but the same issues are common among researchers. For example, Oraby et al., [16] inaccurately claim that “the Danish mask study showed the overall effects from mask wearing and social distancing were modest.” This may be further exacerbated by a misheld belief that randomized trials are automatically “gold standard” evidence, leading to unjustified benefit of the doubt on their usefulness and uncritical adoption of their findings. 

## Conclusions

“Nothing” is often the best alternative use of those resources and “something” often means a potentially costly endeavor which may have little (or negative) contribution toward informed decisions. While Dr. Fretheim argues that DANMASK-19 demonstrates that trials involving NPIs are feasible, it did not demonstrate that what is feasible is informative; uninformative trials can increase uncertainty rather than resolve it. The issues with DANMASK-19 are shared with running randomized trials for many NPIs. Because randomized trials are considered by many as unquestionably robust evidence, uninformative trials for complex and important public health interventions are particularly at risk of causing harm. However, that strong trial designs for important questions are often infeasible does not mean we should accept trials of lower quality, nor that other impact evaluation study designs can adequately fill the gap [17]. There simply may not be a feasible trial that can give us the reliable and direct answers we seek. A too-weak “something” can actively obscure the extent of the true uncertainty and impede alternative decision-strategies, such as hedging bets and leaning more on theoretical grounds. In those circumstances it may be preferable to reconsider whether a trial might be of sufficient value to be worth the costs. Sometimes, an honest and frank “nothing” may be the best option we have.

## Data Availability

Not applicable

## Abbreviations

COVID-19: Coronavirus disease 2019
NPI: Non-pharmaceutical interventions

## Footnotes

1 The DANMASK-19 trial included a single question on compliance which was asked only to the messages arm, and the question asked included only the three options: as recommended, predominantly as recommended, and not as recommended, with no option between predominantly as recommended complying and not as recommended. This also did not ask about mask wearing behavior itself, just self-reported protocol compliance. Further, no question was asked about the mask-wearing behaviors of the non-messages arm. As a result, we do not know the differential mask-wearing behavior between the arms.
